# A New Method for the Characterization of Strain-Specific Conformational Stability of Protease-Sensitive and Protease-Resistant PrP^Sc^

**DOI:** 10.1371/journal.pone.0012723

**Published:** 2010-09-14

**Authors:** Laura Pirisinu, Michele Di Bari, Stefano Marcon, Gabriele Vaccari, Claudia D'Agostino, Paola Fazzi, Elena Esposito, Roberta Galeno, Jan Langeveld, Umberto Agrimi, Romolo Nonno

**Affiliations:** 1 Department of Veterinary Public Health and Food Safety, Istituto Superiore di Sanità, Rome, Italy; 2 Department of Cell Biology and Neurosciences, Istituto Superiore di Sanità, Rome, Italy; 3 Central Veterinary Institute, Wageningen UR, Lelystad, The Netherlands; Creighton University, United States of America

## Abstract

Although proteinacious in nature, prions exist as strains with specific self-perpetuating biological properties. Prion strains are thought to be associated with different conformers of PrP^Sc^, a disease-associated isoform of the host-encoded cellular protein (PrP^C^). Molecular strain typing approaches have been developed which rely on the characterization of protease-resistant PrP^Sc^. However, PrP^Sc^ is composed not only of protease-resistant but also of protease-sensitive isoforms. The aim of this work was to develop a protocol for the molecular characterization of both, protease-resistant and protease-sensitive PrP^Sc^ aggregates. We first set up experimental conditions which allowed the most advantageous separation of PrP^C^ and PrP^Sc^ by means of differential centrifugation. The conformational solubility and stability assay (CSSA) was then developed by measuring PrP^Sc^ solubility as a function of increased exposure to GdnHCl. Brain homogenates from voles infected with human and sheep prion isolates were analysed by CSSA and showed strain-specific conformational stabilities, with mean [GdnHCl]_1/2_ values ranging from 1.6 M for MM2 sCJD to 2.1 for scrapie and to 2.8 M for MM1/MV1 sCJD and E200K gCJD. Interestingly, the rank order of [GdnHCl]_1/2_ values observed in the human and sheep isolates used as inocula closely matched those found following transmission in voles, being MM1 sCJD the most resistant (3.3 M), followed by sheep scrapie (2.2 M) and by MM2 sCJD (1.6 M). In order to test the ability of CSSA to characterise protease-sensitive PrP^Sc^, we analysed sheep isolates of Nor98 and compared them to classical scrapie isolates. In Nor98, insoluble PrP^Sc^ aggregates were mainly protease-sensitive and showed a conformational stability much lower than in classical scrapie. Our results show that CSSA is able to reveal strain-specified PrP^Sc^ conformational stabilities of protease-resistant and protease-sensitive PrP^Sc^ and that it is a valuable tool for strain typing in natural hosts, such as humans and sheep.

## Introduction

Transmissible spongiform encephalopathies (TSEs), or prion diseases, are neurodegenerative disorders that afflict humans and others mammals. TSEs may have genetic, infectious, or sporadic origins. Creutzfeldt-Jakob disease (CJD) is the most common TSE in humans and may be sporadic (sCJD), genetic (gCJD), or acquired (iatrogenic CJD). A novel human acquired prion disease, variant CJD (vCJD), appeared from 1995 onwards and was postulated to be caused by consumption of beef from cows infected with bovine spongiform encephalopathy (BSE). The most common forms of TSE in animals, scrapie in small ruminants, BSE in cattle and chronic wasting disease (CWD) in deer, are all acquired. New atypical forms of BSE in cattle, namely BSE-H [1] and BSE-L or BASE [2] and atypical scrapie in small ruminants, namely Nor98 [3], are supposed to be sporadic.

All TSEs are characterised by the accumulation of PrP^Sc^, a misfolded form of the cellular protein PrP^C^. Besides differing in conformation, the two forms of the prion protein have divergent physical properties: while PrP^C^ is soluble in nondenaturating detergents, rapidly digested by proteases, and rich in α-helical structure, PrP^Sc^ is insoluble in detergents, partially resistant to proteolysis and contains mostly β-sheet [4], [5], [6].

A major problem for the protein-only hypothesis, which postulates that prions are composed mainly or exclusively of PrP^Sc^ [7], was to explain the existence of multiple strains of prions. Prion strains are infectious isolates that, when propagated in the same host, exhibit distinct prion disease phenotypes that persist upon serial transmission [8], [9], [10]. Thus, prion strains cannot be encoded by differences in PrP primary structure but anyway carry information that are independent from the host [11]. The prion hypothesis equates strains to different self-propagating conformational variants of PrP^Sc^ [12].

Several studies demonstrated that prion strains can be distinguished based on different biochemical properties of PrP^Sc^, thus allowing a molecular strain typing approach to TSEs. These studies are based on the electrophoretic features of the protease resistant core of PrP^Sc^ [13], [14], [15], the relative proteinase K resistance of PrP^Sc^ [16], [17], [18], or the physico-chemical behaviour of PrP^Sc^ during denaturation [19], [20] and strongly support the view that distinct strains show different PrP^Sc^ conformations.

A conformational stability assay (CSA), combining guanidine hydrochloride (GdnHCl) denaturation with limited proteolysis using proteinase K, showed that different prion strains may exhibit distinct denaturation profiles [20]. A conformation-dependent immunoassay (CDI) showed the existence of multiple strain-specified PrP^Sc^ conformers by quantifying the immunoreactivity of native and denatured PrP^Sc^ of eight hamster prion isolates [19]. This technique also showed that prion-infected brains contain both protease-sensitive (sPrP^Sc^) and protease-resistant PrP^Sc^ (rPrP^Sc^). More recently, it was shown by CDI that sPrP^Sc^ represents as much as 90% of total PrP^Sc^ in the brain of patients with sporadic CJD [21].

These findings along with the recent discovery in humans [22] and animals [3] of previously undetected TSEs characterised by relatively protease sensitive PrP^Sc^, highlights the need of molecular strain typing methods able to recognize PrP^Sc^ populations based on their physical properties rather than only based on protease digestion.

In this study we aimed at developing a new conformational stability assay based on the differential solubility of PrP^C^ and PrP^Sc^ [23], [24], that we called CSSA (conformational stability and solubility assay). We have previously transmitted CJD and scrapie isolates to bank voles [25], [26], which showed to be a valuable tool for the biological characterization of the most common forms of sCJD, gCJD and scrapie. We took advantage of these vole-adapted strains in order to evaluate the potential of CSSA for strain discrimination.

We first set up experimental conditions allowing the most advantageous separation of PrP^C^ and PrP^Sc^, and thus performed the conformational stability assay by measuring PrP^Sc^ solubility in homogenates treated with increasing concentrations of GdnHCl in the absence of proteinase K. Indeed, insoluble PrP was inversely correlated to GdnHCl concentration, and dose-response curves allowed estimation of the concentration of GdnHCl able to solubilise 50% of PrP^Sc^.

Additionally, we extended the study to natural isolates of sheep scrapie and human sCJD cases to investigate the potential of CSSA for strain discrimination in natural hosts. Our results show that this method is valuable for the biochemical typing of strains in voles and it is also a promising tool for molecular analysis of natural prion isolates.

## Results

### PrP species in healthy and diseased brain

It has been previously reported that normal PrP is composed of full-length PrP (FL-PrP) as well as of 2 C-terminal fragments derived from physiological cleavage at the α and β sites: C1, which is the most represented, derives from α PrP cleavage at position 111/112, while C2 is usually barely detectable and is cleaved around the octarepeat region [27]. Interestingly, α cleavage disrupts the conserved neurotoxic and amyloidogenic region comprising residues 106–126 of PrP, preventing the generation of PrP^Sc^, while β cleavage occurs upstream this conserved region. Accordingly, C2 was reported to be enriched in diseased brains and insoluble in nondenaturing detergents, similarly to PrP^res^ [28].

**Figure 1:**
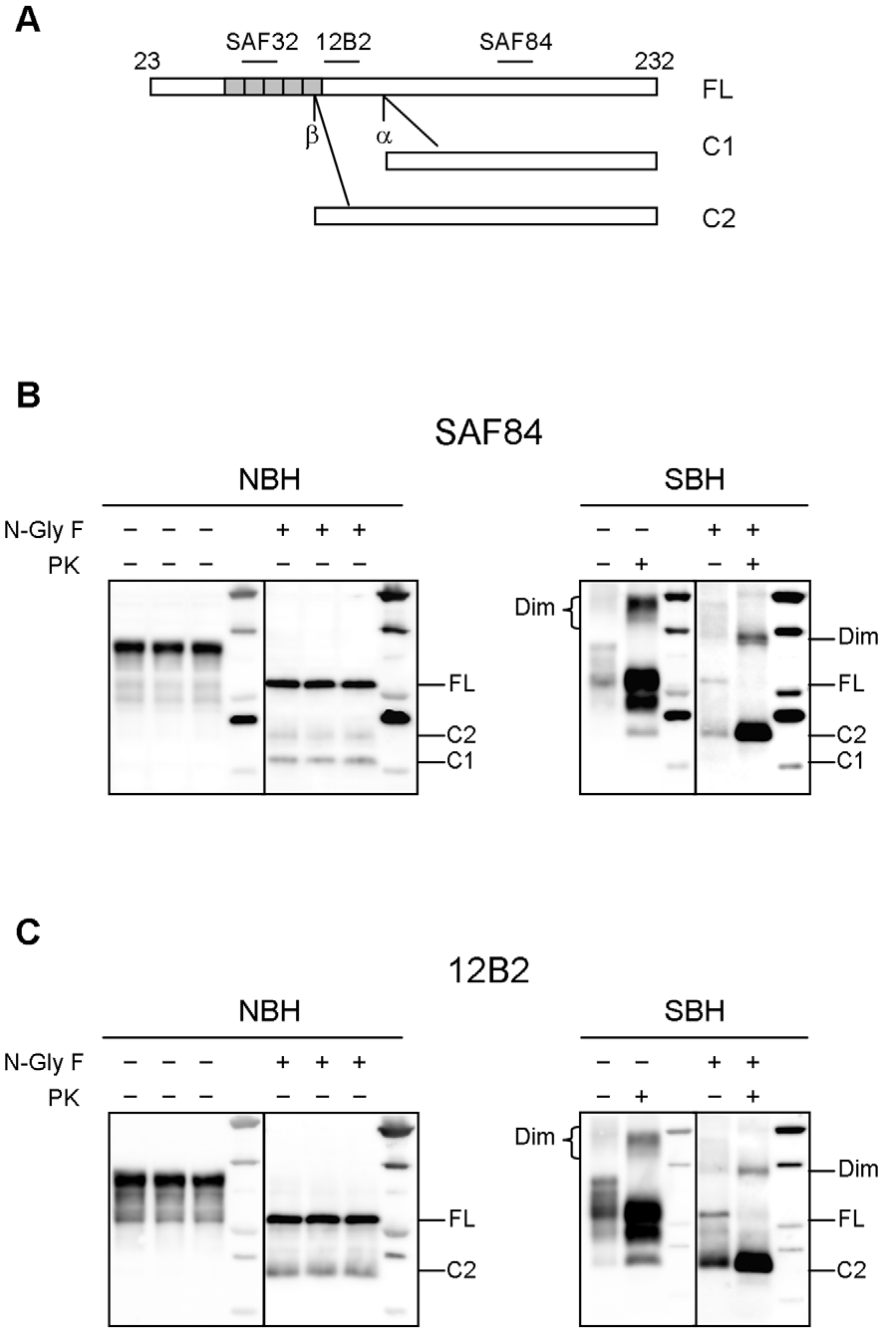
PrP species in healthy and scrapie-affected voles. **A**: Schematic representation of full length PrP (FL) and PrP fragments (C1 and C2) generated by α and β cleavages. The location of SAF84, 12B2 and SAF32 epitopes used for the FL/C1/C2 discrimination are shown. **B** and **C**: Normal brain homogenates (NBH) and scrapie brain homogenates (SBH) of voles were analyzed by western blot using SAF84 (**B**) and 12B2 (**C**). The samples, were analyzed either before or after deglycosylation (N-Gly F) as indicated. The brackets on the left indicate the position of glycosylated and unglycosylated bands of FL, C2 and C1: from 35 kDa to 27 kDa for FL, from 26 kDa to 18 kDa for C2 and from 24 kDa to 16 kDa for C1. These PrP species are reduced to single unglycosylated PrP bands after deglycosylation, which are indicated by dashes on the right of the blots. In SBH, PrPSc dimers (Dim) are also indicated. In NBH both C1 and C2 were present, although C2 was poorly represented; in contrast in SBH C2 fragment was the most abundant PrP species while C1 was barely detectable. Tissue equivalents (TE) loaded per lane were 0,15 mg and 0.5 mg for samples respectively before and after PK digestion. Molecular weight markers were loaded into the last lane of each blot. The positions of MW markers are 15, 20, 25, 37 and 50 kDa.

In order to determine if C1 and C2 were detectable in voles, we analysed healthy and diseased brain homogenates either before or after deglycosylation, with SAF84, whose epitope is present in both fragments, and 12B2 which recognises an epitope that is present only in C2 (Fig. 1A). In normal brain homogenate (NBH), FL-PrP was accompanied by substantial amounts of C1 and lower amounts of C2. In contrast, in voles infected with the Italian scrapie isolate SS7 (SBH) [26] C2 was strongly enhanced while C1 was not easily detectable (Fig. 1B and C). Furthermore, the PrP^res^ fragment generated after PK digestion in SBH was similar to C2 (Fig. 1B and C). Finally, in SBH PrP dimers were present which were completely absent in NBH.

### Separation of PrP^C^ and PrP^Sc^

In order to develop a conformational stability assay based on the differential solubility of PrP^C^ and PrP^Sc^ we first set up the experimental conditions which enabled the most advantageous separation of PrP^C^ from PrP^Sc^. This was obtained through a conventional procedure based on centrifugation in the presence of detergents.

By varying concentrations of different detergents, times of centrifugation and centrifugal force (see Material and Methods), we found that treatment with 2% sarcosyl followed by centrifugation at 20.000 g for 1 h enabled an optimal separation of PrP^C^ from PrP^Sc^.

**Figure 2:**
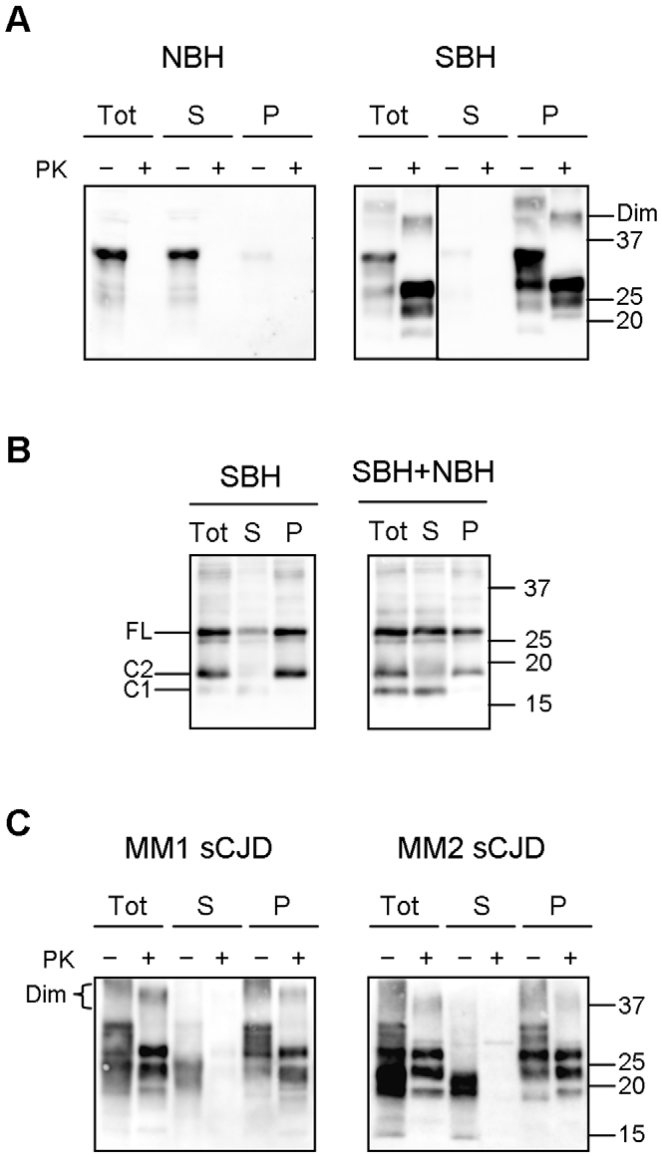
Separation of PrP^C^ and PrP^Sc^ in vole brain homogenates. **A**: Western blot analysis of soluble and insoluble PrP fractions from normal brain homogenate (NBH) and scrapie brain homogenate (SBH) of voles. Samples were centrifuged at 20000 g for 1 h in presence of 2% sarcosyl, and supernatants (S) and pellets (P) were analysed before (+) and after (−) PK treatment. Aliquots of samples before centrifugation (Tot) were analysed too. TE per lane were 0,2 mg for “Tot” and “S” lanes, and 0.4 mg for “P” lanes. Brackets on the left indicate the position of the FL, C1 and C2 PrP fragments. Dimers (Dim) of PrP^Sc^ are indicated on the right in SBH blot. **B**: Western blot analysis of soluble and insoluble PrP fractions from scrapie brain homogenate (SBH) and an artificially mixed sample (SBH+NBH). Samples were centrifuged as described and total (Tot), supernatant (S) and pellet (P) fractions were deglycosylated. Full-length PrP (FL), C1 and C2 PrP fragments are indicated on the left. In each lane, 0.02 mg TE were loaded. **C**: Western blot analysis of soluble and insoluble PrP from brain homogenates of voles infected with MM1 and MM2 sCJD. The samples were treated as in panel A and total (Tot), supernatant (S) and pellet (P) fractions were analysed with or without PK treatment. In each lane 0.3 mg TE were loaded. Brackets on the left indicate the position of the FL, C1 and C2 fragments. Dimers (Dim) of PrP^Sc^ are indicated on the left. **A–C**: Membranes were probed with SAF84. Molecular size markers are shown in kilodaltons on the right of each panel.

In these conditions, >95% of the total PrP^C^ in NBH was soluble and was found in the supernatant fraction (Fig. 2A, left panel) while in SBH most of PrP was sedimented (Fig. 2A, right panel). Unfractionated SBH contained high amounts of PrP^res^ which exclusively sedimented to the pellet fraction (Fig.2A). Insoluble PrP from SBH was indeed mostly PK-resistant (∼90% of insoluble PrP), while soluble PrP was PK-sensitive. All fractions from NBH were devoid of PrP^res^ (Fig. 2A).

Furthermore, soluble and insoluble PrP fractions from SBH displayed slightly different banding patterns which suggested that C2 was mainly insoluble, while soluble PrP contained FL-PrP and the C1 fragment (Fig. 2A). Moreover, PrP^Sc^ dimers were clearly segregated in the insoluble fraction (Fig. 2A). The differential solubility of C1 and C2 in SBH was confirmed by the analysis of deglycosylated PrP species (Fig. 2B), which showed that C1 was completely soluble while C2 was mainly sedimented in the pellet fraction (compare “S” and “P” lanes in Fig. 2B, left panel). With the aim to mimic a situation comparable to that expected in animals with pre-clinical disease, we also studied the differential solubility of C1 and C2 after mixing equal amounts of NBH and SBH (Fig. 2B). Indeed, under these conditions the PrP^C^ content is increased compared to SBH, as can be seen by the higher proportion of C1 in mixed NBH/SBH compared to SBH alone (compare “Tot” lanes in the two panels of Fig. 2B). Also in this condition, C1 was completely soluble and the pellet fraction was enriched in C2 (compare “S” and “P” lanes in Fig. 2B, right panel).

Finally, we investigated the efficacy of our solubility assay for separating PrP^C^ and PrP^Sc^ in voles infected with other, non scrapie-derived prion strains (Fig. 2C). For these experiments we used voles infected with MM1 and MM2 sCJD [25]. In both strains a substantial amount of PrP was found in the insoluble fraction after detergent treatment and centrifugation. As already observed in SBH, the banding patterns of insoluble and soluble PrP were distinct, suggesting a specific precipitation of PrP species associated to disease, namely C2 and PrP dimers (compare “S” and “P” lanes in Fig. 2C). Furthermore, after PK digestion, PrP^res^ was strongly enriched in the pellets and virtually absent in the supernatants.

Collectively, these findings strongly suggest that under the experimental conditions described above we were able to specifically precipitate PrP^Sc^ in brain homogenates from voles infected with different prion strains.

### Conformational stability and solubility assay (CSSA)

The near complete separation of PrP^C^ from PrP^Sc^ allowed us to develop a procedure for biochemical strain typing based on the conformational stability of PrP^Sc^ after exposure to GdnHCl. The conformational solubility assay was set up by measuring PrP^Sc^ solubility in positive brain homogenates treated for 1 hour with increasing concentrations of GdnHCl.

**Figure 3:**
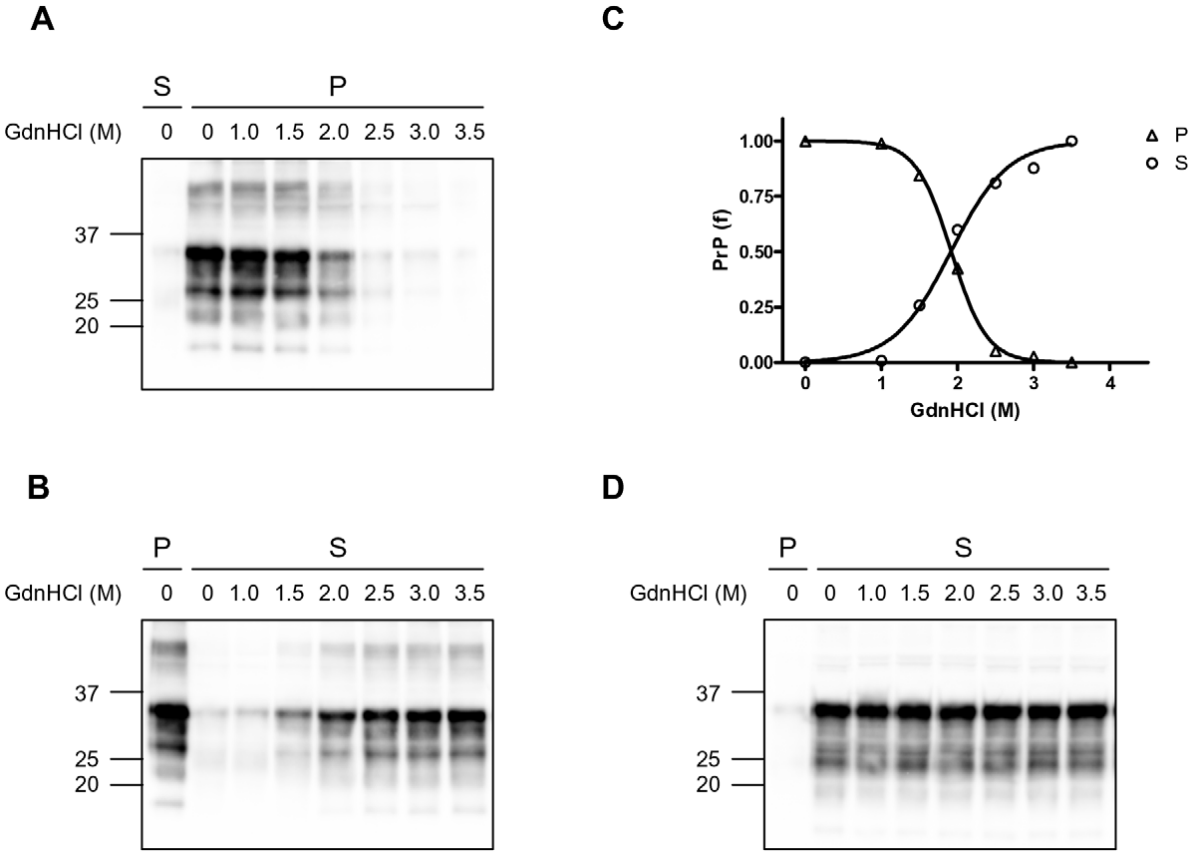
Conformational stability and solubility assay in healthy and diseased voles. **A** and **B**: Western blot analysis of scrapie brain homogenate (SBH) after denaturation with various concentrations of GdnHCl and separation of insoluble (**A**) and soluble (**B**) fractions by centrifugation. **A**: Immunoblot of pellets (P) at different concentrations of GdnHCl. In order to compare the fractions in the same blot, supernatant (S) at 0 M GdnHCl was loaded too. In the pellets, insoluble PrP decreased with increasing concentrations of GdnHCl (M). In each lane 0.4 mg TE were loaded. **B**: Immunoblot of supernatants at different concentrations of GdnHCl. In the first lane pellet (P) at 0 M GdnHCl was loaded too. In the supernatant PrP increased with increasing GdnHCl. In each lane 0.4 mg TE were loaded. Note that the FL and C1 PrP fragments are present in the supernatant after treatment with 0 M and 1 M GdnHCl, while the PrPSc-specific fragment C2 is visible in the supernatant from 1.5 M GdnHCl onwards, in parallel with the decrease of insoluble PrP in panel A. **C**: The conformational stability of PrP^Sc^ in SBH was analysed by denaturation curves best-fitted by plotting the fraction of PrP^Sc^ in the pellet (P) and in the supernatant (S), depicted in panel A and B respectively, as a function of GdnHCl concentration. [GdnHCl]1/2 values were 1.92 M in the pellet and 1.91 M in the supernatant fraction. **D**: Western blot analysis of supernatants (S) at different concentrations of GdnHCl from normal brain homogenate (NBH). In the first lane pellet (P) at 0 M GdnHCl was loaded. In NBH, PrP^C^ was mostly in supernatant fraction and remained soluble at all GdnHCl concentrations tested. Samples were loaded as 0.4 mg tissue equivalent into each lane. **A**, **B**, **D**: Membranes were probed with SAF84. Molecular size markers are shown in kilodaltons on the left of each panel.

As expected, in SBH PrP^Sc^ was solubilized by increasing GdnHCl concentrations (Fig. 3A and B). Indeed, with concentrations of GdnHCl equal or greater than 1.5 M, PrP^Sc^ was partially solubilised and was progressively found in the supernatant instead of the pellet (compare Fig. 3A and 3B). With 3.5 M GdnHCl, virtually all PrP from SBH was found in the soluble fraction (Fig. 3A and 3B). The solubilization of PrP^Sc^ was guanidine-dependent and dose-response curves enabled estimation of the concentration of GdnHCl able to solubilise 50% of PrP^Sc^ ([GdnHCl]_1/2_). The [GdnHCl]_1/2_ value was similar when estimated either in the pellet or in the supernatant fractions (Fig. 3C). In contrast, PrP from NBH remained soluble within the range of GdnHCl concentrations tested (Fig. 3D). Denaturation of PrP^Sc^ was complete after incubation with GdnHCl for 1 hour, as very similar denaturation curves were obtained when the treatment was extended up to 4 hours (Fig. S1). When the denaturation curves where measured in replica blots with mAbs recognising different PrP species, namely SAF32 and SAF84 (see scheme in Fig. 1A), we obtained similar [GdnHCl]_1/2_ values, which suggest that C2 and FL PrP^Sc^ share the same conformational stability (Fig. S2).

**Figure 4:**
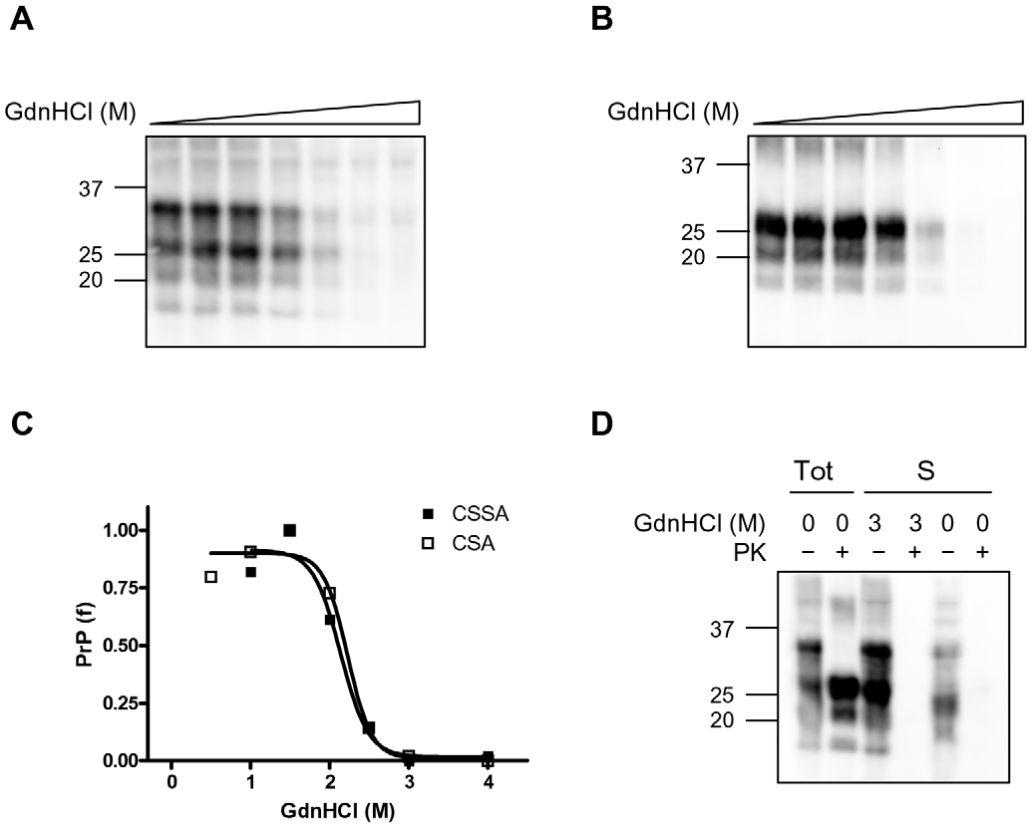
Relationship between PrP^Sc^ insolubility and resistance to PK. The same SBH was analyzed in parallel by CSSA (**A**) and CSA (**B**), and the denaturation curves obtained were compared (**C**). **A** and **B**: Western blot analysis of insoluble PrP^Sc^ (**A**) and PK-resistant PrP^Sc^ (**B**) after denaturation of homogenates with increasing concentrations of GdnHCl. In each lane 0.2 mg TE were loaded. The concentrations of GdnHCl were: 0.5, 1.0, 1.5, 2.0, 2.5, 3.0 and 4.0 M. **C**: Graph depicting the denaturation curves obtained by CSSA (panel A) and CSA (panel B). [GdnHCl]1/2 values were 2.14 M and 2.23 M for CSSA and CSA, respectively. **D**: Western blot showing that PrP^Sc^ loses PK-resistance upon solubilization. In absence of treatment with GdnHCl (0), total PrP (Tot) from SBH was resistant to PK digestion (compare PK- and PK+ lanes). After treatment with 3 M GdnHCl (3) and differential centrifugation, most of PrP^Sc^ was found in the supernatant (S) and was also made PK-susceptible. In absence of treatment with GdnHCl (0), the supernatant (S) contained normal PrP which was susceptible to PK. Note the different banding patterns in the supernatants containing PrP^C^ (0) and solubilised PrP^Sc^ (3). **A**, **B**, **D**: Membranes were probed with SAF84. Molecular size markers are shown in kilodaltons on the left of each panel.

We then studied the relationship between insolubility and PK-resistance of PrP^Sc^ during denaturation. In order to investigate if denaturation equally affects insolubility and PK-resistance, we compared denaturation curves derived from the same SBH, either obtained by insoluble PrP^Sc^ (CSSA) or by PK-resistant PrP^Sc^ (CSA). As shown in Fig. 4, the curves of insoluble PrP^Sc^ (Fig. 4A) and PrP^res^ (Fig. 4B) didn't show differences (Fig. 4C), suggesting that insolubility and PK-resistance were equally susceptible to denaturation by GdnHCl. This finding was further confirmed in experiments aimed at investigating whether, after denaturation, solubilized PrP^Sc^ could partially preserve its resistance to proteinase K. Indeed, after denaturation with 3 M GdnHCl, soluble PrP was fully susceptible to protease digestion (Fig. 4D).

### Conformational stability of scrapie, sCJD and gCJD PrP^Sc^ from bank voles

Under the experimental conditions described above, we investigated the potential of CSSA for differentiating prion strains.

As reported in previous studies [25], [26] sCJD, gCJD and some scrapie isolates present distinct and specific patterns of transmission in voles, based on survival times, lesion profiles, PrP^Sc^ deposition and PrP^res^ biochemical properties. We have shown that voles infected with MM1/MV1 sCJD and E200K gCJD isolates were characterised by a PrP^res^ fragment of ∼19 kDa, MM2 sCJD showed a PrP^res^ fragment of ∼17 kDa, while natural scrapie isolates and murine scrapie ME7 were characterised by a PrP^res^ fragment of ∼18 kDa, intermediate between types 1 and 2 CJD.

Since these PrP^Sc^ types showed specific molecular characteristics after PK digestion, we investigated the conformational stability of PrP^Sc^ in voles infected with MM1 (n = 4), MV1 (n = 3) and MM2 (n = 5) sCJD, E200K gCJD (n = 3) and scrapie SS7 (n = 4).

**Figure 5:**
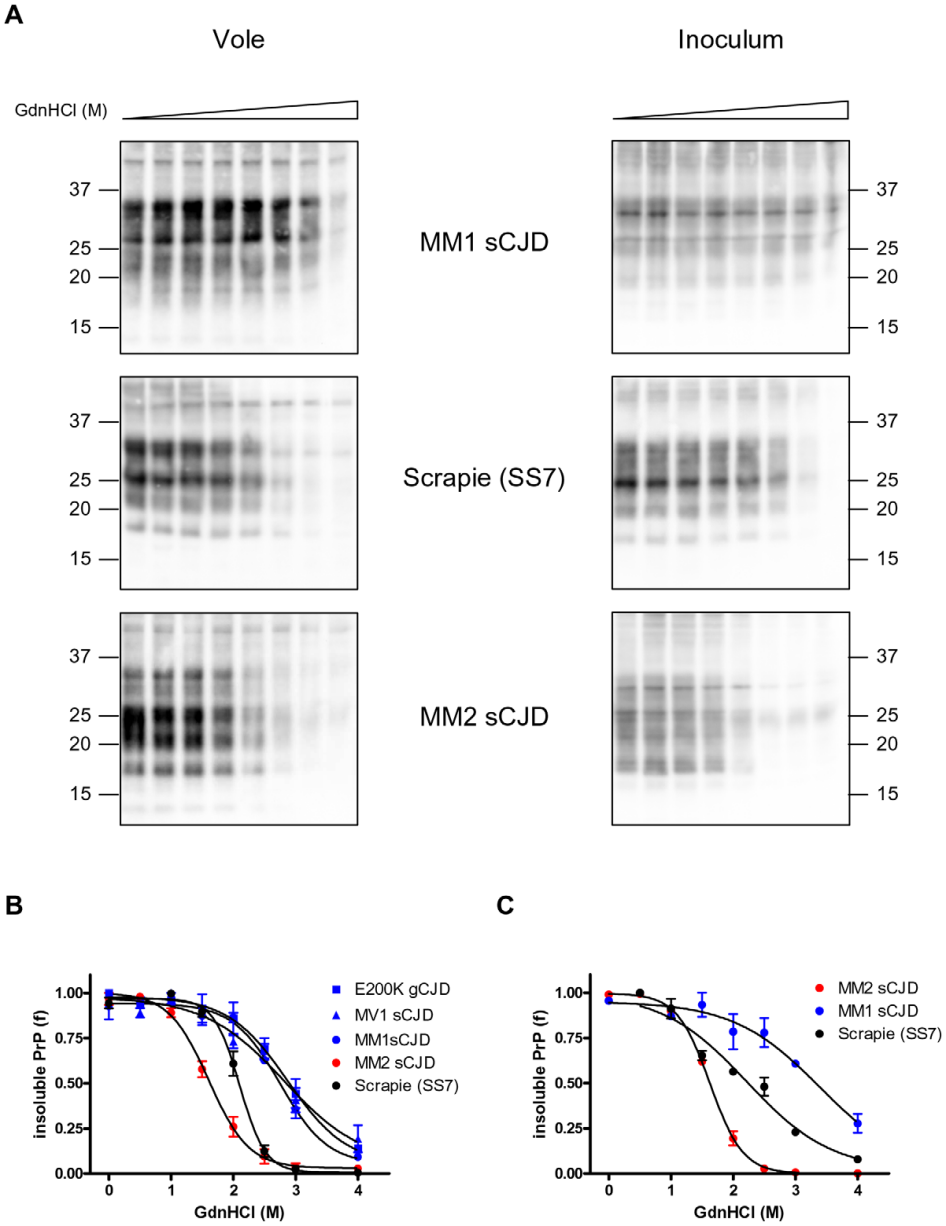
Conformational stability and solubility assay of different strains in voles and natural hosts. **A** and **B**: Conformational stability of PrP^Sc^ from voles inoculated with MM1 sCJD, Scrapie and MM2 sCJD and from the respective human and sheep isolates. **A**: Representative western blots of insoluble PrP^Sc^ from voles (left column) and natural hosts (on the right column) after treatment of homogenates with increasing concentrations of GdnHCl. The concentrations of GdnHCl were: 0, 0.5, 1.0, 1.5, 2.0, 2.5, 3.0 and 4.0 M. TE loaded per lane were 0.2 mg for vole samples, 0.34 mg for human isolates and 0.125 mg for the scrapie isolate. Membranes were probed with SAF84 (vole samples and sheep scrapie) or with L42 (human isolates). **B**: Dose-response curves in vole strains, obtained by plotting the fraction of PrP^Sc^ remaining in the pellet as a function of GdnHCl concentration and best-fitted and using a four parameter logistic equation. Individual curves were combined within each strain group (Scrapie, E200K gCJD, MV1, MM1 and MM2 sCJD) in order to compare the denaturation profiles of the 5 groups. **C**: Dose-response curves from sheep and human isolates, obtained by plotting the fraction of PrP^Sc^ remaining in the pellet as a function of GdnHCl concentration and best-fitted and using a four parameter logistic equation. [GdnHCl]_1/2_ values of MM1, MM2 and scrapie isolates were 3,31 M, 1,63 M and 2,23 M respectively.

Interestingly, CSSA revealed distinct denaturation profiles (Fig. 5), with [GdnHCl]_1/2_ values ranging from 1.5 M to 3 M (Table 1). Voles infected with MM1 sCJD, MV1 sCJD and E200K gCJD showed the highest resistance to denaturation, with mean [GdnHCl]_1/2_ values of 2.77 M, 2.88 M and 2.88 M, respectively. In contrast, PrP^Sc^ from voles infected with MM2 sCJD was the most sensitive (mean [GdnHCl]_1/2_ value of 1.63 M), while scrapie infected voles showed intermediate [GdnHCl]_1/2_ values (2.10 M). We then combined the individual curves within each group in order to compare the denaturation profiles of the 5 groups (Fig. 5B). Scrapie SS7 ([GdnHCl]_1/2_ value of 2.10±0.02) and MM2 sCJD ([GdnHCl]_1/2_ value of 1.61±0.03) showed strain specific denaturation profiles, being significantly different from all other groups (SS7 *vs* MV1, P = 0.0037; SS7 *vs* MM1, P<0.0001; SS7 *vs* E200K, P<0.0001; SS7 *vs* MM2, P<0.0001; MM2 *vs* MV1, P = 0.0022; MM2 *vs* MM1, P<0.0001; MM2 *vs* E200K, P<0.0001). In contrast, MM1 sCJD, MV1 sCJD and E200K gCJD were not significantly different among them (shared [GdnHCl]_1/2_ value of 2.79±0.06, P = 0.52) possibly representing a distinct strain, in agreement with previous findings which showed comparable phenotypes of disease in voles infected with these 3 isolates [25].

**Table 1:** Conformational stability of PrP^Sc^ in vole-adapted sCJD, gCJD and scrapie.

Inoculum	Vole ID	[GdnHCl]_1/2_ (M) ± SEM	[GdnHCl]_1/2_ (M) (mean ± SD)
MM1 sCJD	304/2	2.73±0.17	2.77±0.13
	304/4	2.63±0.12	
	304/8	2.76±0.11	
	304/14	2.94±0.19	
MV1 sCJD	272/1	3.05±0.29	2.88±0.17
	272/4	2.87±0.36	
	272/8	2.72±0.11	
MM2 sCJD	270/6	1.45±0.01	1.63±0.11
	270/7	1.71±0.07	
	350/3	1.60±0.09	
	350/5	1.69±0.04	
	350/14	1.69±0.06	
E200K gCJD	271/4	2.73±0.15	2.88±0.13
	271/5	2.99±0.25	
	271/6	2,91±0.18	
scrapie SS7	388/7	2.22±0.04	2.10±0.13
	388/9	2.16±0.03	
	388/10	1.92±0.02	
	388/11	2.08±0.03	

### Conformational stability of human and sheep isolates

Since we were interested in exploiting our conformational solubility assay (CSSA) for strain discrimination in natural prion diseases, we analysed 3 of the isolates used as inocula for transmission to voles, namely the human MM1 and MM2 sCJD isolates and the sheep scrapie SS7.

At first we tested the efficiency of separation of PrP^Sc^ from PrP^C^ in human and sheep brain homogenates, under the same experimental conditions used for vole brain homogenates. In all samples, PrP^Sc^ was enriched in the pellet, as showed by the segregation of PrP^res^ in the insoluble fraction and by the distinct banding patterns shown by soluble and insoluble PrP (Fig. S3). In negative sheep brain homogenates the fraction of PrP segregating in the pellet was somewhat higher than in vole brains (>10% in some experiment). However, this sedimented PrP^C^ was insensitive to GdnHCl and thus did not interfere with our assay (Fig. S4).

When tested by CSSA, MM1 sCJD, MM2 sCJD and SS7 scrapie revealed different susceptibilities to denaturation (Fig. 5A and B). Interestingly, the rank order of conformational stability and the [GdnHCl]_1/2_ values observed closely matched those found in the vole counterpart, being MM1 sCJD the most resistant ([GdnHCl]_1/2_ = 3.31 M), followed by scrapie SS7 ([GdnHCl]_1/2_ = 2.23 M) and MM2 sCJD ([GdnHCl]_1/2_ = 1.63 M).

### Conformational stability of classical and Nor98 scrapie isolates from sheep

**Figure 6:**
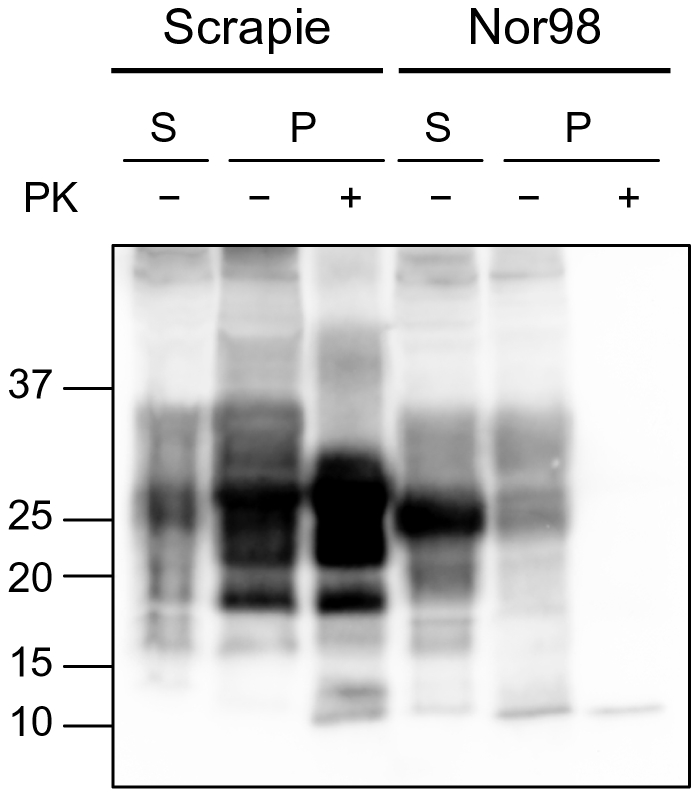
Separation of soluble and insoluble PrP fractions in classical scrapie and Nor98. Western blot analysis of classical scrapie and Nor98 ovine isolates. Supernatant (S) and pellet (P) fractions were analysed with (+) or without (−) PK treatment. In each lane 0.8 mg TE were loaded. Molecular size markers are shown in kilodaltons on the left of the blot. Membrane was probed with L42.

In order to test the ability of CSSA for studying the conformational stability of protease-sensitive PrP^Sc^, we took advantage of the recently described atypical scrapie strain, Nor98 [3], which has been shown to induce the accumulation of relatively protease-sensitive PrP^Sc^ [29]. As previously observed for all other strains investigated, insoluble and soluble Nor98 PrP^Sc^ showed different segregation of PrP species (Fig. 6). Interestingly the 12 kDa PrP^res^ fragment, which is a characteristic feature of Nor98 PrP^res^ [3], [30], was observed before PK-digestion and segregated with insoluble PrP, in a manner similar to what was observed for C2 in all other strains. However, in Nor98 only a minimal fraction of insoluble PrP was protease resistant (Fig. 6), while PrP^res^ represented >90% of insoluble PrP in classical scrapie (Fig. 6). These findings confirm that in Nor98 samples disease-associated PrP is mostly PK-sensitive, although accompanied by low amounts of genuine PK-resistant PrP^Sc^.

**Figure 7:**
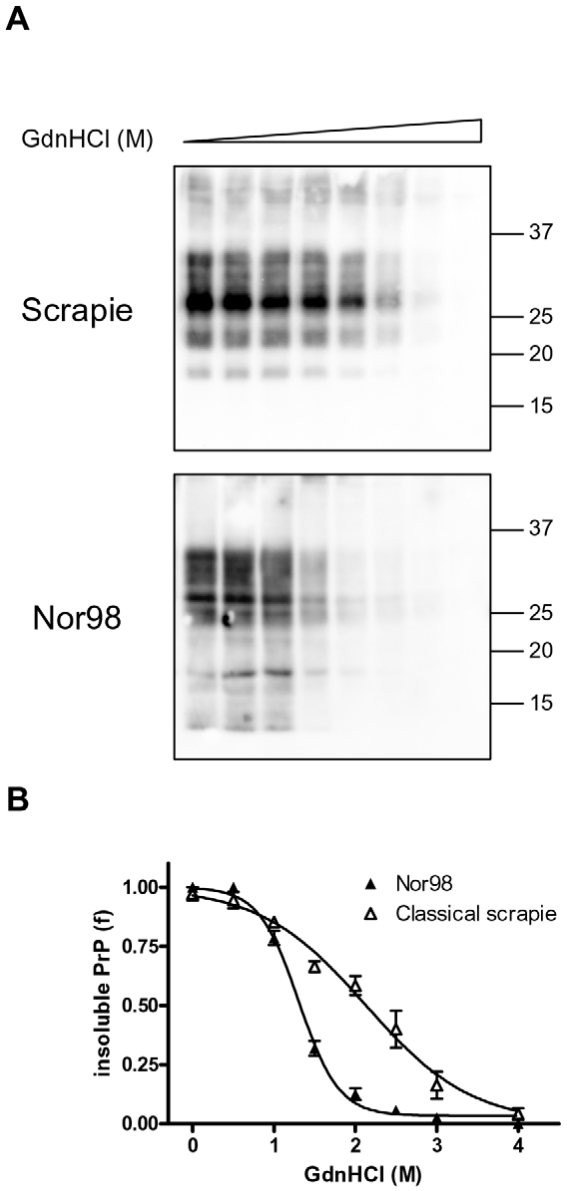
CSSA of classical scrapie and Nor98 isolates. **A**: Representative western blots of insoluble PrP after denaturation with increasing concentrations of GdnHCl in classical scrapie (ARQ/AHQ) and Nor98 (ARR/AHQ) isolates. The concentrations of GdnHCl were: 0, 0.5, 1.0, 1.5, 2.0, 2.5, 3.0 and 4.0 M. TE loaded per lane were 0.125 mg for classical scrapie and 0.3 mg for Nor98. Membranes were probed with L42. Molecular size markers are shown in kilodaltons on the right of each blot. **B**: Dose-response curves of Nor98 and classical scrapie, obtained by plotting the fraction of PrP^Sc^ remaining in the pellet as a function of GdnHCl concentration and best-fitted and using a four parameter logistic equation. Individual curves were combined within each strain group (Scrapie and Nopr98) in order to compare the denaturation profiles of the two groups. Individual [GdnHCl]_1/2_ values are shown in Table 2.

We then studied the conformational stability of Nor98 (n = 5) and classical (n = 4) Italian field scrapie cases of different PrP genotypes (Table 2). Classical scrapie samples gave denaturation curves very similar to that of SS7, with [GdnHCl]_1/2_ values ranging from 1.96 to 2.31 (Fig. 7 and Table 2). Nor98 samples gave remarkably similar denaturation profiles, independently of PrP genotype, and showed high sensitivity to GdnHCl denaturation (Fig. 7), with [GdnHCl]_1/2_ values ranging from 1.26 to 1.43 (Table 2). Within group comparison didn't show significant differences among classical scrapie (shared [GdnHCl]_1/2_ value of 2.09±0.11, P = 0.52) and Nor98 (shared [GdnHCl]_1/2_ value of 1.36±0.04, P = 0.50). Comparison of the combined classical scrapie and Nor98 denaturation profiles (Fig. 7B) gave statistically significant different [GdnHCl]_1/2_ values (P<0.0001).

**Table 2:** Conformational stability of PrP^Sc^ in sheep field prion isolates.

Strain	Sheep ID	Age (y)	Clinical signs	PrP Genotype*	[GdnHCl]_1/2_ (M)	[GdnHCl]_1/2_ (M) (mean±SD)
Classical scrapie	ES8/09/3	2	Yes	ALRQ/ALRQ	2.27	2.20±0.16
	ES8/09/1	6	No	ALRQ/ALHQ	1.96	
	241/105	3	No	ALRQ/ALRQ	2.27	
	211/26	3	Yes	ALRQ/ALRQ	2.31	
Nor98	ES36/08/4	12	No	ALHQ/ALRR	1.26	1.32±0.07
	ES35/07/2	9	No	ALRQ/ALHQ	1.26	
	ES10/07/7	12	No	AFRQ/AFRQ	1.35	
	ES18/07/2	5	No	ALRQ/ALHQ	1.31	
	ES19/07/23	7	No	ALRR/ALRR	1.43	

*amino acids at codons 136, 141, 154 and 171.

## Discussion

The main pathogenetic event in TSEs involves the trans-conformation of PrP^C^ to PrP^Sc^, which leads to the accumulation of detergent-insoluble and partially protease-resistant aggregates. Thus, resistance to PK digestion and insolubility are the hallmarks of PrP^Sc^. However, previous studies on the molecular characterization of PrP^Sc^ were mainly focused on the PK-resistant core of PrP^Sc^, due to the difficulties in differentiating PrP^C^ from PrP^Sc^ in infected brain homogenates. Notwithstanding, it is becoming increasingly clear that insoluble but protease-sensitive isoforms of PrP are involved in different animal and human prion diseases [19], [22], [31], [32], [33], [34], [35]. These PrP isoforms have been detected by several methods, including conformation-dependent immunoassay [19], [21], [33], immunological capture [36], [37], differential centrifugation [31], [32], thermolysin digestion [35] and cold PK digestion [34].

Among the above-mentioned techniques able to detect sPrP^Sc^, the CDI was also reported to distinguish eight hamster prion strains based on PrP^Sc^ conformation, by plotting the ratio of antibody binding to denatured/native PrP as a function of the concentration of PrP^Sc^ [19]. However, CDI depends on the availability of monoclonal antibodies able to recognise buried epitopes in PrP^Sc^, such as the 3F4. We developed a protocol for the molecular characterization of PrP^Sc^ aggregates which does not rely on their protease resistance but is based on a conventional procedure of differential centrifugation in the presence of detergents and on the solubilization of PrP^Sc^ aggregates upon denaturation with increasing concentrations of GdnHCl. Our findings show that CSSA is reliable and straightforward, and that it is able to discriminate PrP^Sc^ conformers associated with different TSE strains. Compared to CDI, we believe that CSSA offer some interesting advantages: i) it does not depend on the antibody used and can be thus exploited to compare PrP^Sc^ conformational stability in different species, ii) the proof that CDI is able to discriminate prion strains in natural hosts is still lacking, while here we show the potential of CSSA to be used in human and sheep natural isolates.

In previous studies [20], [38], [39] a conformational stability assay (CSA) able to discriminate PrP^Sc^ conformers was set up by measuring the extent of loss in protease resistance as a function of increased exposure to GdnHCl. This method proved to be very helpful for molecular strain typing in different species [40], [41], [42] and was also recently exploited for investigating some basic mechanisms of prion replication [39]. The protocol that we developed is conceptually similar to CSA, as it derives information on the conformational stability of PrP^Sc^ aggregates. Indeed, we showed that CSA and CSSA gave very similar denaturation profiles in vole-adapted scrapie brain homogenates (see Fig. 4). However, we believe that CSSA represents a step forward in prion molecular typing and offers several advantages compared with previously used protocols. With CSA, in fact, susceptibility to PK is exploited to distinguish denaturated from native PrP^Sc^. However, different prion strains may have distinct susceptibilities to PK, while CSA uses the same PK concentration to derive the level of PrP^Sc^ denaturation in different strains. Furthermore, when performing CSA it is necessary to dilute the GdnHCl to allow the activity of proteolytic enzyme. However, it is known that PrP^Sc^ unfolding can be a partially reversible phenomenon and, as reported, the dilution of the denaturant could restore the original protease-resistance of PrP^Sc^ [43]. With CSSA this problem was circumvented by avoiding any change in denaturant concentration during the assay. Indeed, the denaturation step is followed by the centrifugal separation of soluble and insoluble fractions, which is performed under conditions identical to the denaturation step.

Most importantly, CSSA allowed the characterization of protease-sensitive PrP^Sc^, and thus the direct comparison of the conformational stabilities of TSE strains associated with protease-sensitive and protease-resistant PrP^Sc^. We investigated the potential of CSSA for strain typing of ovine field isolates of Nor98, which are characterised by high amounts of insoluble sPrP^Sc^ [29, present paper]. Our findings seem very encouraging, since Nor98 samples displayed a distinct denaturation profile, although the isolates derived from sheep of various PrP genotypes and presumably at different stage of the disease. Furthermore, Nor98 samples were easily discriminated from classical scrapie, which represents a different strain. Thus CSSA enabled characterization of the conformational stability of a protease sensitive strain, for which methods based on proteolysis would have characterised only a minimal amount of PrP^Sc^ present in the brain homogenate. This is promising in view of recent studies [21], [35] showing that in sCJD and vCJD isolates, as much as 90% of PrP^Sc^ in the brains was estimated to be sPrP^Sc^. Furthermore the recent discovery of protease sensitive prionopathy (PSPr), a previously unrecognised human prion disease [22], might suggest that prion diseases characterised by protease-sensitive PrP isoforms are more frequent than previously thought. Brain homogenates from PSPr cases were reported to contain mainly a protease sensitive form of insoluble PrP, accompanied by low amounts of typical protease-resistant PrP [22], similarly to our findings in Nor98 samples. It would be interesting to investigate the potential of CSSA for characterising these human protease-sensitive disease-related isoforms of PrP.

Another interesting feature of CSSA is that, by avoiding PK treatment, it allowed the characterization of FL-PrP^Sc^, including the N-terminus which is cleaved upon PK digestion. Besides FL-PrP, *in vivo* trimmed PrP^Sc^ fragments were selectively precipitated and thus CSSA also conveyed information on their conformational stability. These fragments comprised C2 C-terminal fragments in scrapie and sCJD isolates, as well as the 12 kDa internal fragment characteristic of Nor98, and may be easily distinguished from FL-PrP, also by means of differential antibody binding (data not shown; see for example Fig. S2). This is interesting because it enables a comparison of the conformational properties of PrP^Sc^ aggregates made-up of FL-PrP or *in vivo* trimmed PrP^Sc^ fragments. *In situ* epitope-mapping of PrP^Sc^, indeed, showed that FL and C2 PrP^Sc^ aggregates have different cellular localizations [44]. Furthermore, by analysing thermolysin-resistant PrP^Sc^ in sheep scrapie and BSE isolates, Owen and colleagues have recently shown the potential of C2 fragments for strain typing [45].

The main potential drawback of CSSA might be the incomplete separation or PrP^C^ and PrP^Sc^. Herein, we developed a protocol able to minimise this problem, notwithstanding 1–4% of PrP^C^ in brain homogenates from healthy voles was found in the pellet under our working conditions. There are however several lines of evidence suggesting that this potential drawback did not interfere with CSSA results. We have shown that brain homogenates from clinically affected voles contain low levels of soluble PrP (*bona fide* PrP^C^) (see Fig. 2). Based on the results obtained with NBH, less than 5% of PrP^C^ is expected to be found in the pellet; on the other hand, in diseased brains *bona fide* PrP^C^ was 5–30% of total PrP. From these considerations, it can be argued that the PrP^Sc^/PrP^C^ ratio in the pellet should be higher than 2 orders of magnitude, which represents the working range of CSSA. Furthermore, we obtained clear-cut differential solubility of PrP^C^ and PrP^Sc^ even after increasing the PrP^C^/PrP^Sc^ ratio in the homogenate, by mixing SBH with NBH (Fig. 2). Most importantly, we found that the sedimented PrP^C^ fraction was insensitive to denaturation (Fig. S4) and thus it does not interfere with CSSA.

We investigated the potential of CSSA for strain discrimination by analysing vole-adapted strains deriving from human sCJD, gCJD and sheep scrapie which we have previously shown to give distinct strains in voles [25], [26]. In previous studies we have shown that voles infected with MM1/MV1 sCJD and E200K gCJD isolates showed identical transmission patterns and were characterised by an unglycosylated PrP^res^ fragment of ∼19 kDa, while MM2 sCJD showed an unglycosylated PrP^res^ fragment of ∼17 kDa. In contrast, all Italian scrapie isolates studied so far [26], [46], as well as the murine scrapie strain ME7 [25], were characterised by the accumulation of an unglycosylated PrP^res^ with a molecular weight (∼18 kDa) intermediate between CJD types 1 and 2, upon transmission to voles. These different PrP^res^ types derive from different PK-cleavage sites of PrP^Sc^, which in turn are believed to reflect distinct conformations of PrP^Sc^ aggregates. With CSSA we showed that these three PrP^res^ types are actually characterised by PrP^Sc^ aggregates with distinct susceptibilities to denaturation by GdnHCl. Indeed, PrP^Sc^ from voles infected with MM1 sCJD, MV1 sCJD and E200K gCJD, characterised by 19 kDa PrP^res^, showed the highest resistance to denaturation, while sCJD MM2, characterised by 17 kDa PrP^res^, was the most susceptible and scrapie SS7, that was characterised by 18 kDa PrP^res^, displayed intermediate susceptibility. Interestingly, the within group variability of [GdnHCl]_1/2_ values was very low (see table 1) and allowed statistical comparisons among the different groups, which strengthened the view that [GdnHCl]_1/2_ values reflect strain-specific rather than individual PrP^Sc^ properties.

It has been recently suggested that the conformational stability of PrP^Sc^ is directly proportional to the length of the incubation time in mice [39]. On this point, it is worth noting that our results in voles, although based on only 5 isolates, seem to contradict this conclusion. Indeed, the lowest conformational stability was associated with prions with the longest survival times, i.e. MM2 sCJD with a survival time of ∼330 days post-inoculation (dpi), while scrapie SS7 (survival time of ∼90 dpi) and MM1/MV1 sCJD (survival time of ∼130 dpi) showed higher conformational stabilities and shorter survival times compared to MM2 sCJD. Further studies with an extended panel of isolates are needed to investigate if the direct relationship between conformational stability and incubation time observed in mice holds true also for vole prions.

We also explored the potential of CSSA for strain typing of natural prion diseases. To this aim we analysed three of the human and sheep isolates which were used for bioassay in vole. Interestingly, we found that the two isolates from MM1 sCJD and MM2 sCJD patients could be easily discriminated by their conformational stabilities, with MM1 sCJD displaying lower susceptibility to denaturation compared to MM2 sCJD ([GdnHCl]_1/2_ values of 3,31 M for MM1 sCJD and 1,63 M for MM2 sCJD). These findings compare well with previous studies by CSA, which showed that PrP^Sc^ associated with MM1 sCJD was ∼ 2-fold more stable than that of MM2 sCJD, with [GdnHCl]_1/2_ values of 2,76 M and 1,63 M for MM1 sCJD and MM2 sCJD [40]. Of course, this needs confirmation in a larger set of isolates.

Furthermore, this approach allowed us to compare the conformational stability of prions in their natural host and after transmission to voles. It has been previously shown that prion strains can either maintain their biological properties or mutate upon propagation in a new host species [10]. More recently it was reported that a change in conformation was accompanied by the emergence of a new prion strain during interspecies transmission, while the conformational stability of PrP^Sc^ was preserved when a strain “bred true” in the new host [38]. Our findings show that the rank order of conformational stability of sCJD and scrapie isolates was generally preserved upon adaptation in voles (see Fig. 5). In particular, the conformational stability of MM2 sCJD was identical before and after transmission in voles. We have also previously reported that MM2 sCJD, and to a lower extent MM1 sCJD, encountered a very low transmission barrier to voles when compared to several other prion strains [25]. These observations may suggest that MM2 and possibly MM1 sCJD faithfully propagated their strain properties upon transmission in voles. The “conformational selection” model, postulates that host PrP^C^ primary structure influences which of the portfolio of possible PrP^Sc^ types are thermodynamically preferred during propagation [47]. In this model, the transmission barrier is determined by the degree of overlap between the subset of PrP^Sc^ types allowed or preferred by PrP^C^ in the host and donor species. It can be thus speculated that the vole PrP sequence is prone to adopt some human PrP^Sc^ conformations and we have previously shown that this property may reside in the presence of some peculiar amino acid at relevant positions of vole PrP [46], [48].

Finally, we exploited CSSA for discriminating sheep field prion isolates, showing that this method is valuable for strain discrimination in natural hosts. Indeed, the conformational stability of prions was strongly associated with the strain, either classical scrapie or Nor98, and did not depend on individual properties such as age, clinical stage or PrP genotype (see table 2). Although preliminary, these findings suggest that CSSA might be exploited as complementary approach for increasing the discriminatory power of strain typing in small ruminant TSEs. Studies with natural and experimental TSEs, including sheep BSE and CH1641-like isolates, are underway.

## Materials and Methods

### Terminology

Throughout the manuscript we used “prion” to refer to the infectious agent of TSEs, “PrP^Sc^” to refer to the abnormal disease-associated PrP isoform, “rPrP^Sc^” to refer to the protease-resistant fraction of PrP^Sc^, “sPrP^Sc^“ to refer to the protease-sensitive fraction of PrP^Sc^ and “PrP^res^” to refer to protease-resistant PrP^Sc^ fragments deriving from rPrP^Sc^ trimming by proteinase K.

### Natural TSE isolates

Human brain tissues were from cerebral cortices of two sCJD cases (MM1 and MM2 types) previously characterised by vole bioassay [25].

The medulla oblongata of SS7 scrapie was obtained from an ARQ/ARQ Sarda sheep with clinical scrapie reported in Italy in 1997 and previously characterised by vole bioassay [26]. All others sheep brain tissues were from Italian field cases detected by active and passive surveillance and characterised by discriminatory western blotting for molecular strain typing and by complete sequencing of the PRNP open reading frame, according to published protocols [49], [50]. The details of these TSE cases are reported in Table 2.

### Vole samples

Brain tissues were obtained from voles infected with vole-adapted human and sheep TSEs as previously reported [25], [26]. The passage number and mean survival times ± standard deviation of the vole groups from which the samples used in the present study derived were as follows: sCJD MM1, second passage, 129±8 dpi; sCJD MV1, second passage, 128±15 dpi; sCJD MM2, second passage, 339±27 dpi; sCJD MM2, third passage, 323±41 dpi; gCJD E200K, second passage, 143±12 dpi; scrapie SS7, third passage, 93±5 dpi.

### Separation of PrP^Sc^ and PrP^C^ by differential centrifugation

Brain homogenates (20% w/v) were prepared in 100 mM Tris-HCl with Complete protease inhibitor cocktail [Roche] at pH 7.4. The homogenates were either used directly or stored at −20°C.

The experimental conditions for PrP^C^/PrP^Sc^ separation were set up in vole brain homogenates by studying the effect of different detergents, centrifugal force and time of centrifugation.

Brain homogenates (3% to 12% w/v) were added with equal volumes of different buffers (100 mM Tris-HCl at pH 7.4 containing Sarcosyl 4% or 2%; 100 mM Tris-HCl at pH 7.4 containing NaDoc 1%, NP40 1%; 100 mM Tris-HCl at pH 7.4 containing Triton X-100 2%) and incubated for 1 hour at 37°C with gentle shaking. Then samples were centrifuged at 10000 to 20000 g for 1 h or 2 h. The obtained pellets were re-suspended with 100 mM Tris-HCl (pH 7.4) containing the relevant detergent. The experimental conditions then used throughout the paper in vole, sheep or human brain tissues included solubilisation in 100 mM Tris-HCl at pH 7.4 containing sarcosyl 2% and centrifugation at 20000 g for 1 h. For each of the different experimental conditions tested, equivalent aliquots of brain homogenate before centrifugation, along with supernatant and pellet fractions, were analysed by western blot either with or without PK digestion.

### Conformational stability and solubility assay

Aliquots of brain homogenates (3% to 6% w/v) were added with an equal volume of 100 mM Tris-HCl (pH 7.4) containing sarcosyl 4% and incubated for 1 h at 37°C with gentle shaking. Aliquots of 100 µl were treated with 100 µl of guanidine hydrochloride (GdnHCl) solutions with a final concentration ranging from 0 to 4.0 M. GdnHCl stock solutions were prepared from an 8 M solution (Pierce) diluted in water. After treatment with GdnHCl for 1 h at 37°C with gentle shaking, samples were centrifuged at 20000 g for 1 h at 22°C. Pellets were re-suspended in 90 µl NuPage LDS Sample Buffer (Invitrogen) and 10 µl NuPage Sample Reducing Agent (Invitrogen). Aliquots of supernatants were precipitated with 4-fold volume excess of pre-chilled methanol 30 min at −20°C, centrifuged at 15000 g for 30 min at 4°C and then were re-suspended in 90 µl NuPage LDS Sample Buffer (Invitrogen) and 10 µl NuPage Sample Reducing Agent (Invitrogen). Supernatant and pellet fractions were analysed by Western blotting.

Individual denaturation curves were analyzed and best-fitted by plotting the fraction of PrP^Sc^ remaining in the pellet as a function of GdnHCl concentration, and using a four parameter logistic equation (GraphPad Prism). In order to fit denaturation curves for each prion strain, the mean fraction of PrP^Sc^ remaining in the pellet ± SD were plotted. Statistical comparison of [GdnHCl]_1/2_ values were made by comparing the best-fit value for each data set with GraphPad Prism. This was performed by either fitting each data set independently or doing a global fit with a shared [GdnHCl]_1/2_ value, and then the results were compared with an F test. The simpler model was selected unless the extra sum-of-squares F test had a P value<0.05.

### Digestion with proteinase K after denaturation with GdnHCl

Aliquots of the same brain homogenate were treated in parallel according to CSSA and conformational stability assay (CSA) protocols. The CSA was performed as described [20], with minor modifications. Aliquots of brain homogenates (6% w/v) were added with an equal volume of 100 mM Tris-HCl (pH 7.4) containing sarcosyl 4% and incubated for 1 h at 37°C with gentle shaking. Aliquots of 50 µl were added with 50 µl of GdnHCl to give a final concentration ranging from 0 to 4.0 M. After 1 h of incubation at 37°C all samples were diluted to a final concentration of 0.4 M GdnHCl. Proteinase K (50 µg/ml) was added and the samples were incubated for 1 h at 37°C with gentle shaking. The reaction was stopped with 3 mM PMSF (Sigma). Aliquots of samples were added with an equal volume of isopropanol/butanol (1∶1 v/v) and centrifuged at 20000 g for 5 min. Pellets were re-suspended in NuPage LDS Sample Buffer (Invitrogen) and were analysed by Western Blotting.

### Western blot analysis

Electrophoresis and Western blotting were performed as previously described [25]. Samples were denatured by adding NuPage LDS Sample Buffer (Invitrogen, Carlsbad, California, United States) and NuPage Sample Reducing Agent (Invitrogen), and heating for 10 min at 90°C. After centrifugation at 10000 g for 5 min each sample was loaded onto 12% bis-Tris polyacrylamide gels (Invitrogen). After electrophoresis and Western blotting on PVDF membranes (Immobilon-P; Millipore, Bedford, MA, USA), the blots were processed by SNAP i.d.™ Protein Detection System (Millipore) as described by the manufacturer instructions.

The monoclonal antibodies used, their epitopes on sheep PrP and the working dilutions were as follow: SAF84, PrP residues 167–173, 1.2 µg/ml; L42, PrP residues 148–153, 0.28 µg/ml; 12B2, PrP residues 93–97, 2.4 µg/ml and SAF32, PrP octarepeat, 2.4 µg/ml. Horseradish peroxidase-conjugated anti-mouse immunoglobulin (Pierce Biotechnology, Rockford, Illinois, United States) was used as secondary antibody (1∶13000).

The membranes were developed with an enhanced chemiluminescence method (SuperSignal Femto, Pierce). Chemiluminescence signal was detected with the VersaDoc imaging system (Bio-Rad) and was quantified by QuantityOne software (Bio-Rad).

Deglycosylation was performed by adding 18 µl of 0.2 M sodium phosphate buffer (pH 7.4) containing 0.8% Nonidet P40 (Roche) and 2 µl (80 U/ml) di N-Glycosidase F (Roche) to 5 µl of denaturated samples and by incubating overnight at 37°C with gentle shaking. Samples were then analysed by Western blotting as described above.

## Supporting Information

Figure S1 Effect of denaturation time on CSSA. Dose-response curves of insoluble PrP from brain homogenates of voles infected with scrapie SS7 (top panel) and MM1 sCJD (bottom panel) after treatment with increasing concentrations of GdnHCl for 1, 2 or 4 hours. Denaturation curves were best-fitted by plotting the fraction of PrP remaining in the pellet as a function of GdnHCl concentration. SS7 and MM1 didn't reveal differences based on time of treatment and showed the same [GdnHCl]_1/2_ values at 1, 2 and 4 hours (2.1 M for SS7 e 3 M for MM1 sCJD).
(0.13 MB TIF)

Figure S2 CSSA with different mAbs. A: Representative western blots of insoluble PrP from vole infected with E200K gCJD after denaturation with increasing concentrations of GdnHCl. Replica blots were probed with SAF84 (top) and SAF32 (bottom), as indicated on the left of the blot. The concentrations of GdnHCl were: 0, 0.5, 1.0, 1.5, 2.0, 2.5, 3.0 and 4.0 M. In each lane 0.2 mg TE were loaded. B: Dose-response curves derived from the blots in panel A, obtained by plotting the fraction of PrP remaining in the pellet as a function of GdnHCl concentration and best-fitted and using a four-parameter logistic equation.
(0.57 MB TIF)

Figure S3 Separation of soluble and insoluble PrP fractions from human and sheep isolates. Western blot analysis of soluble and insoluble PrP from brain homogenates of sheep with classical scrapie and human with MM1 sCJD. Samples were centrifuged at 20000 g for 1 h in presence of 2% Sarcosyl, and supernatants (S) and pellets (P) were analysed with (+) or without (−) PK treatment. Aliquots of samples before centrifugation (Tot) were analysed too. TE loaded per lane were 0.2 mg for classical scrapie and 0.15 mg for MM1 sCJD. Scrapie membrane was probed with SAF84 and MM1 sCJD membrane was probed with L42.
(0.48 MB TIF)

Figure S4 Separation of soluble and insoluble PrP fractions from sheep normal brain homogenates. A: Western blot analysis of soluble and insoluble PrP fractions from a representative sheep negative brain homogenate. Total PrP (Tot) and PrP from supernatant (S) and pellet (P) fractions were analysed with (+) or without (−) PK treatment. TE loaded per lane were 0.2 mg. B: Western blot analysis of insoluble PrP from sheep negative and scrapie brain homogenates after denaturation with increasing concentrations of GdnHCl, either after normal (top panel) or long (bottom panel) exposure times. The concentrations of GdnHCl were: 0, 0.5, 1.0, 1.5, 2.0, 2.5, 3.0 and 4.0 M. TE loaded per lane were 0.12 mg. A and B: Membranes were probed with SAF84.
(0.69 MB TIF)
