# Supplementary figures and images for: A New Method for the Characterization of Strain-Specific Conformational Stability of Protease-Sensitive and Protease-Resistant PrPSc
Source: PLoS One. 2010 Sep 14;5(9):e12723. doi: 10.1371/journal.pone.0012723 (PMC2939050; doi:10.1371/journal.pone.0012723)

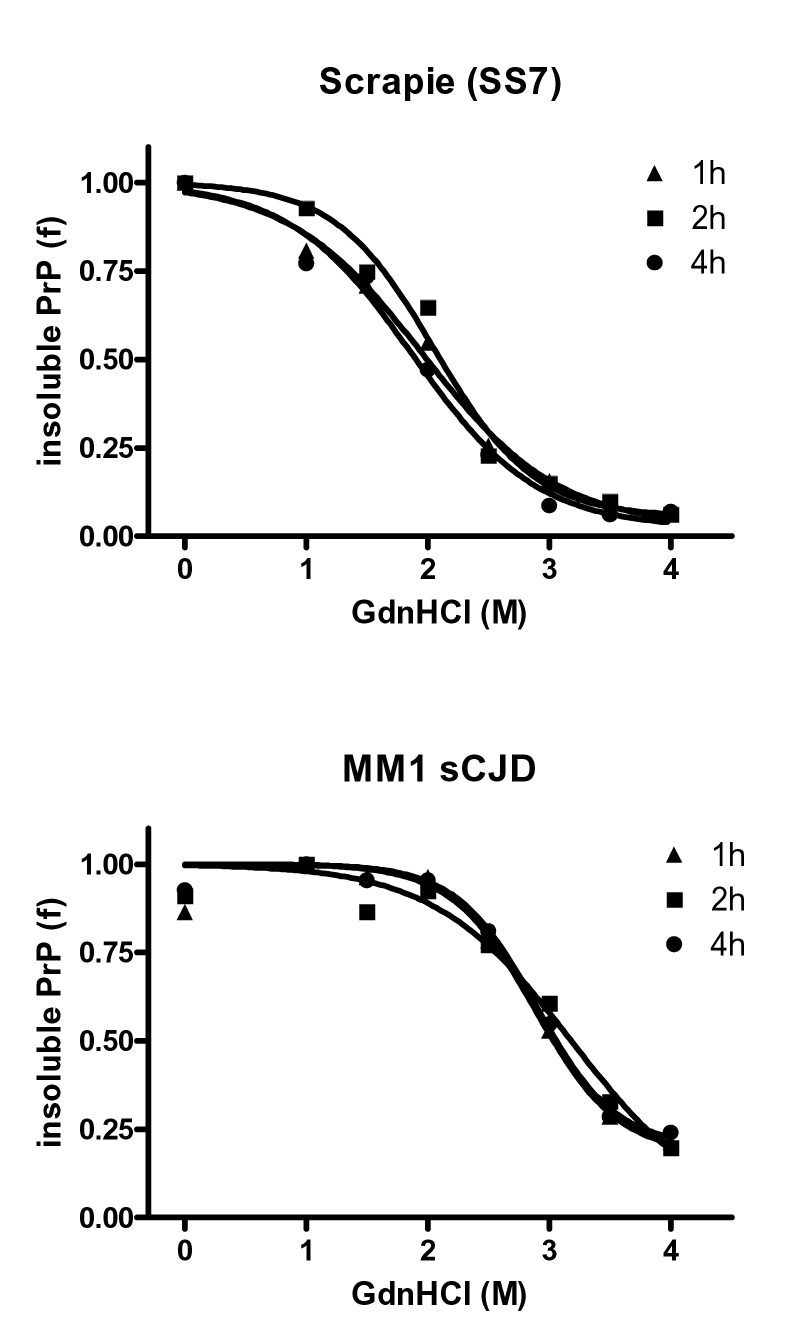

Supplement: Figure S1 — Effect of denaturation time on CSSA. Dose-response curves of insoluble PrP from brain homogenates of voles infected with scrapie SS7 (top panel) and MM1 sCJD (bottom panel) after treatment with increasing concentrations of GdnHCl for 1, 2 or 4 hours. Denaturation curves were best-fitted by plotting the fraction of PrP remaining in the pellet as a function of GdnHCl concentration. SS7 and MM1 didn't reveal differences based on time of treatment and showed the same [GdnHCl]1/2 values at 1, 2 and 4 hours (2.1 M for SS7 e 3 M for MM1 sCJD). (0.13 MB TIF) [file pone.0012723.s001.tif]

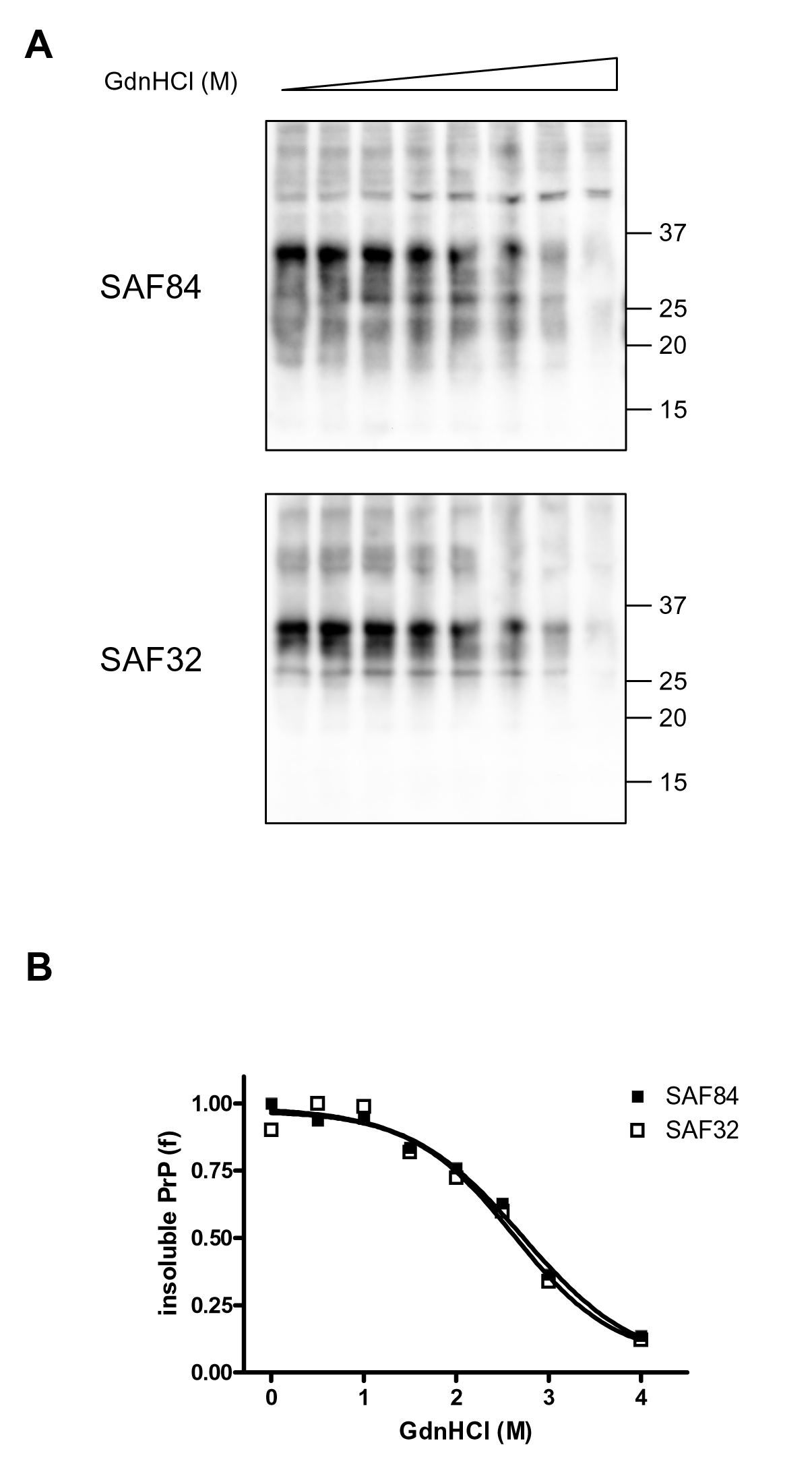

Supplement: Figure S2 — CSSA with different mAbs. A: Representative western blots of insoluble PrP from vole infected with E200K gCJD after denaturation with increasing concentrations of GdnHCl. Replica blots were probed with SAF84 (top) and SAF32 (bottom), as indicated on the left of the blot. The concentrations of GdnHCl were: 0, 0.5, 1.0, 1.5, 2.0, 2.5, 3.0 and 4.0 M. In each lane 0.2 mg TE were loaded. B: Dose-response curves derived from the blots in panel A, obtained by plotting the fraction of PrP remaining in the pellet as a function of GdnHCl concentration and best-fitted and using a four-parameter logistic equation. (0.57 MB TIF) [file pone.0012723.s002.tif]

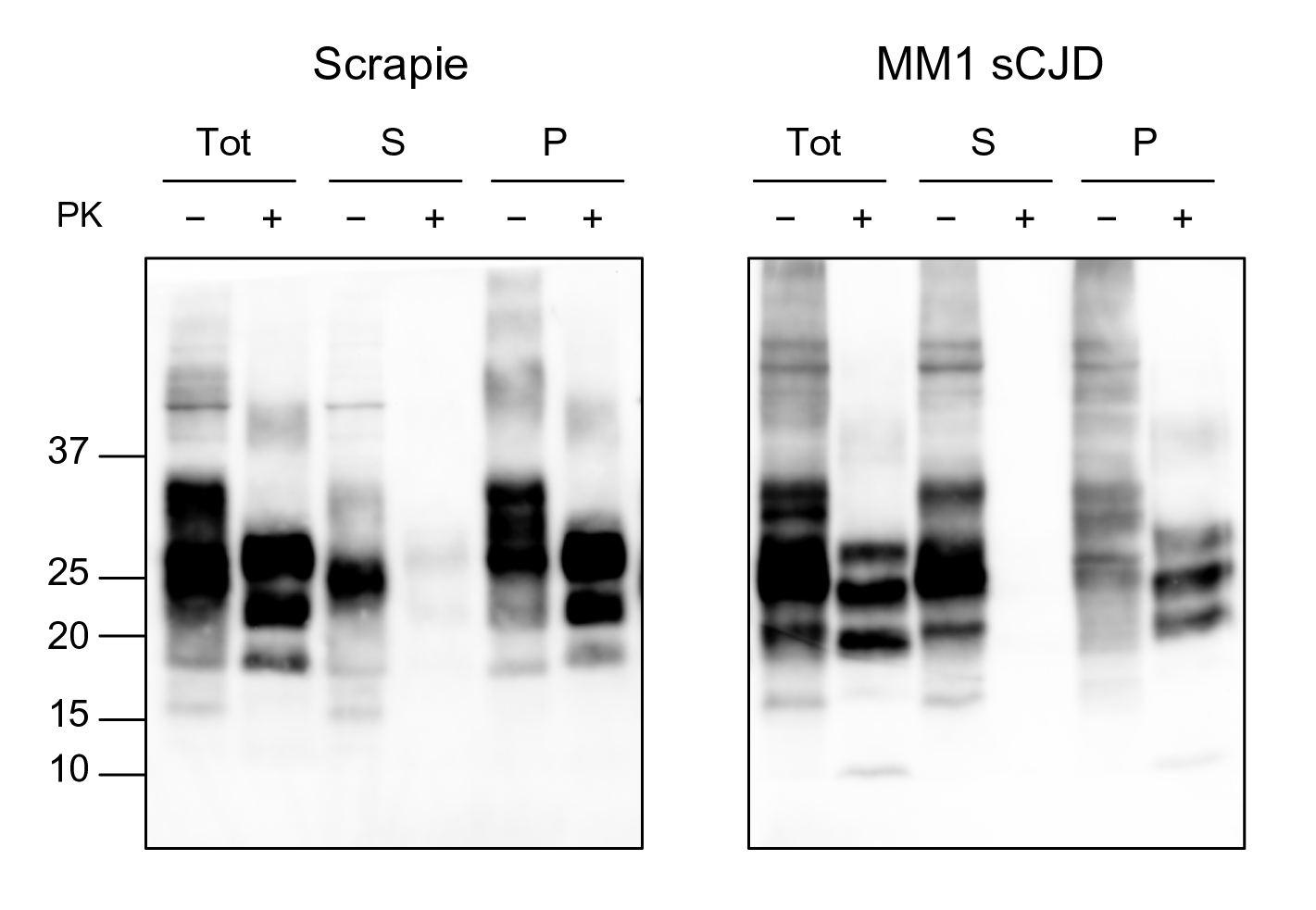

Supplement: Figure S3 — Separation of soluble and insoluble PrP fractions from human and sheep isolates. Western blot analysis of soluble and insoluble PrP from brain homogenates of sheep with classical scrapie and human with MM1 sCJD. Samples were centrifuged at 20000 g for 1 h in presence of 2% Sarcosyl, and supernatants (S) and pellets (P) were analysed with (+) or without (−) PK treatment. Aliquots of samples before centrifugation (Tot) were analysed too. TE loaded per lane were 0.2 mg for classical scrapie and 0.15 mg for MM1 sCJD. Scrapie membrane was probed with SAF84 and MM1 sCJD membrane was probed with L42. (0.48 MB TIF) [file pone.0012723.s003.tif]

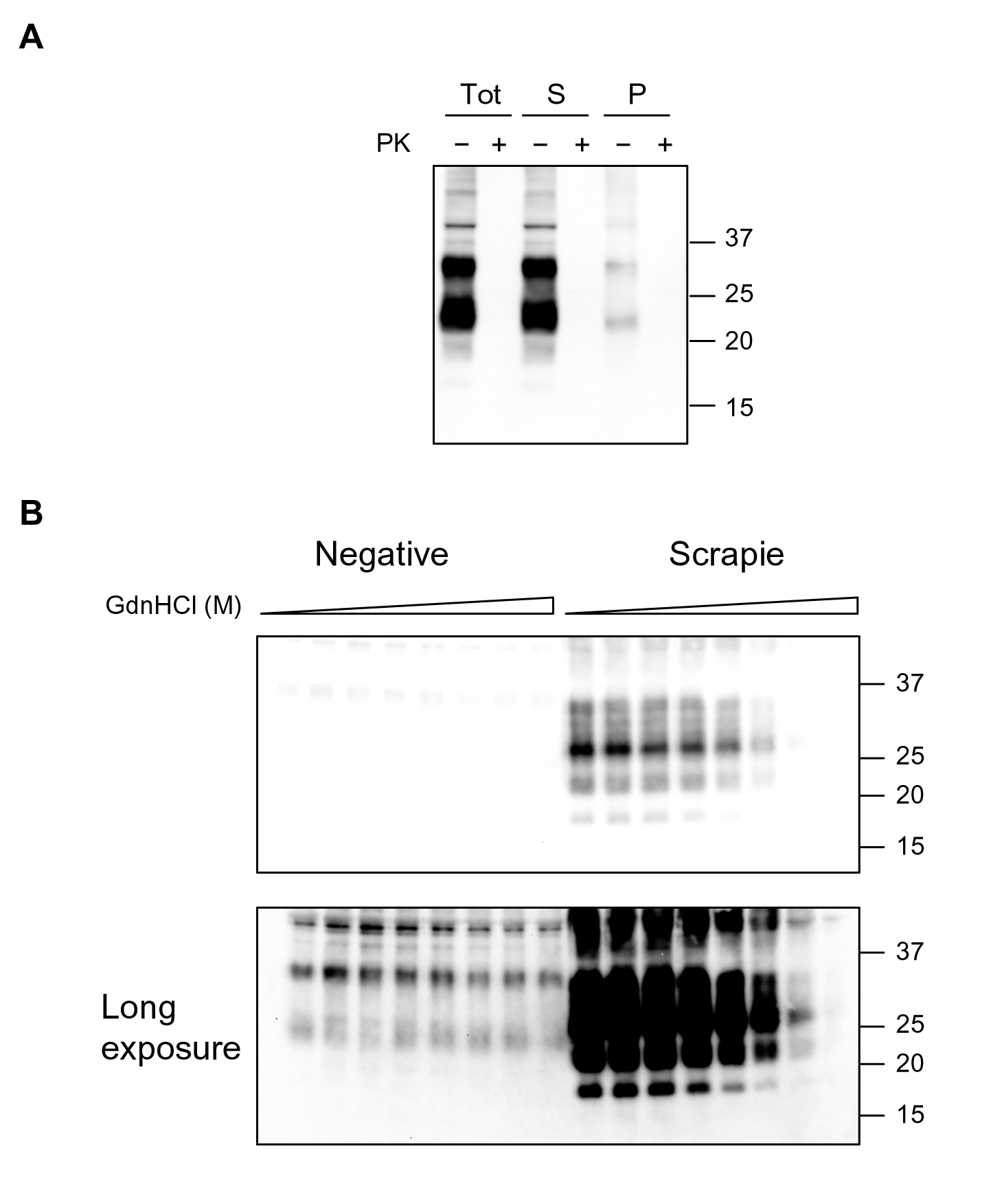

Supplement: Figure S4 — Separation of soluble and insoluble PrP fractions from sheep normal brain homogenates. A: Western blot analysis of soluble and insoluble PrP fractions from a representative sheep negative brain homogenate. Total PrP (Tot) and PrP from supernatant (S) and pellet (P) fractions were analysed with (+) or without (−) PK treatment. TE loaded per lane were 0.2 mg. B: Western blot analysis of insoluble PrP from sheep negative and scrapie brain homogenates after denaturation with increasing concentrations of GdnHCl, either after normal (top panel) or long (bottom panel) exposure times. The concentrations of GdnHCl were: 0, 0.5, 1.0, 1.5, 2.0, 2.5, 3.0 and 4.0 M. TE loaded per lane were 0.12 mg. A and B: Membranes were probed with SAF84. (0.69 MB TIF) [file pone.0012723.s004.tif]
